# Ampullary Carcinoma: Prognostic Factors and a Literature Review

**DOI:** 10.3390/cancers18040707

**Published:** 2026-02-22

**Authors:** Ivánia Furtado, Nuno Gião, Ana Gonçalves, Emanuel Vigia, Mariana Sardinha, João Boavida Ferreira

**Affiliations:** 1 Department of Medical Oncology, Unidade Local de Saúde de São José, 1169-050 Lisbon, Portugal; mariana.sardinha@ulssjose.min-saude.pt (M.S.); joao.ferreira2@ulssjose.min-saude.pt (J.B.F.); 2 Centro Clínico Académico de Lisboa, 1169-056 Lisbon, Portugal; nuno.giao@ulssjose.min-saude.pt (N.G.); ana.carvalho5@ulssjose.min-saude.pt (A.G.); emanuel.duarte@ulssjose.min-saude.pt (E.V.); 3 Department of Anatomical Pathology, Unidade Local de Saúde de São José, 1069-166 Lisbon, Portugal; 4 Department of Surgery, Unidade Local de Saúde de São José, 1069-166 Lisbon, Portugal

**Keywords:** ampullary carcinoma, gastrointestinal neoplasms, histologic subtypes, tumor biomarkers, prognosis, adjuvant therapy, recurrence-free survival, overall survival

## Abstract

Background/Objectives: Ampullary carcinoma (AC) is a rare gastrointestinal malignancy arising from the ampullary complex, encompassing intestinal, pancreaticobiliary, and mixed subtypes with distinct biological behaviors. Surgery is the only potentially curative treatment, yet relapse occurs in more than half of patients. Due to the scarcity of AC-specific prospective trials, treatment guidelines are largely extrapolated from other gastrointestinal cancers. This study aimed to characterize the clinicopathological features, treatment approaches, and outcomes of AC and to identify potential prognostic factors in a single-center cohort. Methods: We retrospectively analyzed 106 patients diagnosed with AC between January 2015 and December 2023 to characterize clinicopathological features, treatment approaches, and survival outcomes, and to explore potential prognostic factors. Kaplan–Meier analysis was used to estimate recurrence-free survival (RFS) and overall survival (OS), while prognostic factors were assessed using univariate and multivariable Cox regression models. Results: Most patients presented with resectable disease and underwent pancreaticoduodenectomy (96.2%). The predominant histological subtype was pancreaticobiliary (45.3%). In localized disease, median RFS and OS were not reached, with 36-month RFS and OS rates of 68.1% (95% CI, 59.2–78.3) and 70.1% (95% CI, 61.1–80.3), respectively. In univariate analyses, adverse prognostic factors for both RFS and OS included the advanced T TNM category, nodal involvement, lymphovascular invasion, perineural invasion, high-grade histology, and R1 resection margins; however, only R1 resection margin remained independently associated with shorter RFS in the multivariate analysis (HR 2.5, 95% CI 1.02–5.94, *p* = 0.046). Survival outcomes did not differ significantly according to histological subtype. Exploratory adjusted analyses accounting for nodal status and surgical resection margin suggested an association between adjuvant therapy and improved survival, while unadjusted analyses showed no significant associations. Median OS for metastatic patients was 13.6 months. Discussion/Conclusion: R1 resection margin emerged as the only independent prognostic factor for RFS, with no independent association with OS, in resected AC. These findings highlight the importance of surgical margin optimization, high-quality pathological assessment, and multidisciplinary management in routine clinical practice.

## Simple Summary

Ampullary carcinoma is a rare cancer that is largely underrepresented in prospective clinical trials. As a result, treatment strategies are often extrapolated from other gastrointestinal tumors, and clear criteria for selecting patients who may benefit from adjuvant therapy are lacking. In this retrospective observational study, we analyzed clinical data from patients with ampullary carcinoma to explore factors associated with survival outcomes. We also reviewed the existing medical literature to place our findings in context. Advanced tumor stage, lymph node involvement, lymphovascular/perineural invasion, high tumor grade, and positive resection margins were associated with worse outcomes; however, only positive resection margins were independently associated with shorter recurrence-free survival, and no factor was independently linked to overall survival. While adjuvant therapy may have a role, improved patient selection is needed to maximize benefit. These findings highlight the need for better risk stratification strategies and the importance of multidisciplinary decision-making in managing this uncommon disease.

## 1. Introduction

Ampullary carcinoma (AC) is a rare gastrointestinal (GI) cancer, accounting for approximately 0.2% of all GI malignancies [1]. Nevertheless, recent data indicate a rising incidence of AC [1,2]. This malignancy originates from the ampullary complex, an anatomical structure comprising the intraduodenal portions of the common bile duct and pancreatic duct, along with the ampulla of Vater [3].

Diagnosis is usually made at an early stage, as biliary obstruction leads to early-onset symptoms, with jaundice being the most common presentation [4,5].

The majority of AC are adenocarcinomas and can be classified into 3 histological subtypes based on their epithelial origin: intestinal type, arising from intestinal epithelium; pancreaticobiliary type, originating from the biliary or pancreatic ductal epithelium; and mixed type, which contains features of both intestinal and pancreaticobiliary subtypes, with each component representing at least 25% of the tumor architecture [2,6]. Subtypes could differ in prognosis, with the intestinal type generally showing better outcomes [1,6]. However, data supporting histological subtype as an independent prognostic factor remain controversial [7].

Due to the lack of robust prospective phase 3 clinical trials specific to AC, current treatment guidelines are largely extrapolated from evidence in colorectal, pancreatic, and biliary cancers, depending on the histological subtype [8].

Surgery is the only potentially curative treatment. Despite the relatively early stage and resectability at diagnosis, more than 50% of patients experience relapse after surgery [7,9]. For resectable disease, lymph node status and surgical resection margins are recognized independent prognostic factors. Other potential prognostic indicators include perineural and lymphovascular invasion, tumor grade, and patient-related factors, including age and Eastern Cooperative Oncology Group performance status (ECOG PS) [10,11]. The role of neoadjuvant and adjuvant treatments is not well defined due to a scarcity of evidence. For unresectable locally advanced or stage IV disease, systemic treatment is indicated, although the optimal treatment regimen remains unclear [7,8].

In this context, we combined a single-center cohort analysis with a contextual literature review to place our institutional findings within the framework of current management strategies for AC. The aim of this study was to characterize the clinical and pathological features, treatment approaches, and outcomes of AC and to explore potential prognostic factors in a cohort of patients diagnosed over an 8-year period.

## 2. Materials and Methods

### 2.1. Study Design and Patient Selection

We conducted a retrospective, single-center study of patients with newly diagnosed AC at our institution between January 2015 and December 2023. Eligible patients had histologically confirmed adenocarcinoma and complete baseline clinical and pathological data. Patients with non-tubular histological patterns (e.g., medullary carcinoma, poorly cohesive cell carcinoma, or undifferentiated carcinoma) or neuroendocrine tumors were excluded. Tumor subtype classification was performed based on hematoxylin–eosin morphology combined with immunohistochemistry (MUC1, MUC2, CDX2, and MUC5AC), according to the World Health Organization Classification of Tumors (5th Edition). All cases were evaluated centrally as part of the routine diagnostic workflow at our tertiary referral center, performed by gastrointestinal pathologists with subspecialty expertise. Tumors initially categorized as “mixed or not specified” were analyzed as a single mixed subtype. In these cases, the “mixed” designation reflected overlapping morphologic and immunophenotypic features consistent with a mixed phenotype. Data were pseudonymized prior to analysis. The study received approval from the Institutional Ethics Committee of Unidade Local de Saúde de São José and was conducted in accordance with the Declaration of Helsinki. Informed consent was waived due to the retrospective design.

### 2.2. Variables and Data Collection

Data on demographics, clinical presentation, imaging findings, pathological characteristics (tumor size, lymph node status, histological subtype and grade, lymphovascular invasion, perineural invasion, and surgical resection margins), and treatment modalities were extracted from electronic health records. Localized disease was defined as clinical stage I–III, whereas metastatic disease was defined as stage IV, according to the AJCC 8th edition. We further assessed prognostic factors associated with survival outcomes, including clinical, pathological, and treatment-related variables.

### 2.3. Objectives and Outcomes Definitions

The objectives were to evaluate recurrence-free survival (RFS) and overall survival (OS) in patients with localized disease, OS and progression-free survival in the metastatic setting (PFSmet), and to identify clinical, pathological, and treatment-related prognostic factors.

OS was defined as the time from diagnosis of AC to death from any cause in patients with localized disease and from the diagnosis of metastatic disease to death in patients with metastatic AC. RFS was defined as the time from curative-intent surgery to the first documented recurrence or death. PFSmet was defined as the time from diagnosis of metastatic AC to the first disease progression or death. For the calculation of OS, RFS, and PFSmet, the cut-off date was set at December 2023, and patients who had not experienced an event by this date were censored. Recurrence and progression were determined based on radiological or pathological evidence. Radiologic recurrence was assessed using standard cross-sectional imaging (computed tomography and/or magnetic resonance imaging) and confirmed in a multidisciplinary setting.

### 2.4. Statistical Analysis

Patient and tumor characteristics were summarized using descriptive statistics. Survival curves were estimated by the Kaplan–Meier method and compared between groups with the log-rank test. Variables with *p* < 0.05 in univariate Cox proportional hazards models were entered into multivariate models to identify independent prognostic factors, with hazard ratios (HRs) and 95% confidence intervals (CIs) reported. Given the limited sample size and number of events, this approach was adopted to minimize model instability and the risk of overfitting. Because adjuvant therapy was not randomly allocated, treatment effect analyses were descriptive and evaluated using parsimonious multivariable Cox regression adjusted for lymph node status and surgical resection margin status. The proportional hazards assumption was assessed using log-minus-log survival plots. Missing data were handled using a complete-case approach, with statistical analyses performed on available data only. All tests were two-sided, and *p* < 0.05 was considered statistically significant. Analyses were performed using SPSS Statistics version 29.0.2.0.

## 3. Results

### 3.1. Demographics and Clinical Characteristics

A total of 106 patients were analyzed. Table 1 provides clinical and demographic details. The median age at diagnosis was 69 years (interquartile range [IQR], 63–77 years), and the majority were male (*n* = 62, 58.5%). Most patients had ECOG PS ≤ 1 (*n* = 98, 92.5%). At diagnosis, 96.2% of patients (*n* = 102) presented with localized, resectable disease, most of which was classified as clinical stage III (*n* = 65, 61.3%). 4 patients (3.8%) had metastatic disease at diagnosis. The pancreaticobiliary subtype was the predominant histological type in the cohort (*n* = 48, 45.3%). The most frequently reported clinical presentation was obstructive jaundice (*n* = 91, 85.8%). Serum tumor marker increased levels (CA 19-9, CEA, or both) were observed in less than half of the patients (*n* = 50, 47.2%).

Surgery with curative intent was performed in 102 patients (96.2%), all with localized disease. 1 patient received neoadjuvant chemotherapy with FOLFIRINOX. Positive resection margins were observed in 13 patients (12.7%), and positive lymph node involvement was present in 62 patients (60.8%). Tumoral lymphovascular and perineural invasion were identified in 55 (53.9%) and 44 (43.1%) patients, respectively. Grade 3 tumors were found in 25 patients (24.5%).

Marked differences were observed between the pancreaticobiliary and intestinal histological subtypes. The pancreaticobiliary subtype displayed a more aggressive pathological profile, with higher frequencies of T3–T4 tumors (65.2% vs. 47.4%), node-positive disease (80.4% vs. 34.2%), lymphovascular invasion (69.6% vs. 42.1%), perineural invasion (63.0% vs. 26.3%), and R1 surgical resection margins (17.4% vs. 10.5%). Histological grade 3 tumors were uncommon in the pancreaticobiliary subtype (4.3%), whereas the intestinal subtype exhibited a higher proportion of high-grade tumors (15.8%).

Adjuvant therapy was administered to 48 patients (47.1%), including 39 (38.2%) who received chemotherapy and 9 (8.8%) who underwent chemoradiotherapy. Gemcitabine and capecitabine were the most commonly used agents in chemotherapy and chemoradiotherapy, respectively. A summary of the treatment regimens is provided in Table 1.

Use of adjuvant therapy was more frequent in the pancreaticobiliary subtype (52.2% vs. 36.8%), and recurrence occurred more often in this group (30% vs. 21.1%), further supporting its overall more aggressive biological behavior.

Among patients with metastatic disease at diagnosis, 3 received palliative systemic treatment: FOLFIRINOX (*n* = 1), FOLFOX (*n* = 1), and gemcitabine (*n* = 1).

### 3.2. Outcomes

The median follow-up for the study cohort was 29.3 months (range, 1–113.6).

#### 3.2.1. Recurrence-Free Survival

**Figure 1:**
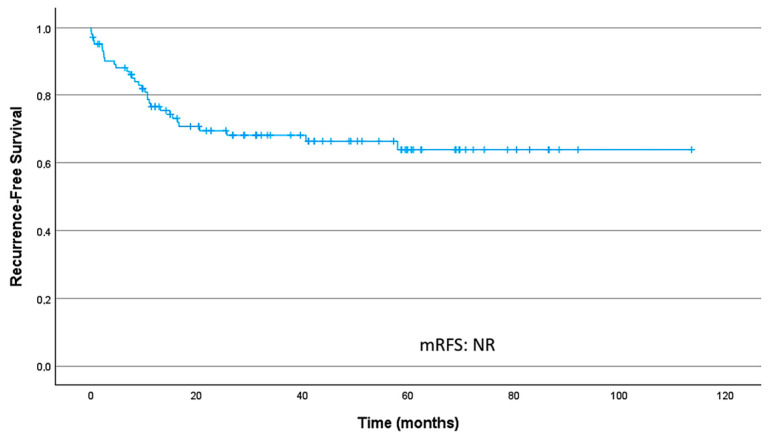
Recurrence-free survival curve in localized disease; median recurrence-free survival (mRFS) was not reached.

In the localized setting, among patients who developed recurrence, the median time from surgery to recurrence was 25.7 months (IQR, 10.1–56.6). The median RFS (mRFS) for the overall population was not reached ([NR], 95% CI NR–NR). RFS rates at 24 and 36 months were 69.4% (95% CI 60.6–79.4) and 68.1% (95% CI 59.2–78.3), respectively. Figure 1 presents the overall RFS curve in localized disease.

#### 3.2.2. Prognostic Factors for Recurrence-Free Survival

In univariate analysis, adverse prognostic factors for RFS included T3–T4 tumors (HR 3.2, 95% CI 1.40–7.07, *p* = 0.003), lymph node involvement (HR 2.35, 95% CI 1.05–5.27, *p* = 0.031), lymphovascular invasion (HR 2.45, 95% CI 1.16–5.29, *p* = 0.015), perineural invasion (HR 2.9, 95% CI 1.44–6.18, *p* 0.002), histological grade 3 tumors (HR 2.7, 95% CI 1.36–5.55, *p* = 0.003), and R1 resection margins (HR 4.06, 95% CI 1.85–8.92, *p* < 0.001), as shown in Table 2.

Obstructive jaundice at diagnosis was associated with a trend toward longer RFS (HR 0.85, 95% CI 0.30–2.40, *p* = 0.77). Perioperative serum tumor marker levels were not associated with RFS (CEA: HR 0.77, 95% CI 0.23–2.56, *p* = 0.853; CA 19-9: HR 1.46, 95% CI 0.72–2.92, *p* = 0.282).

In multivariate analysis, only R1 resection margin remained independently associated with shorter RFS (HR 2.5, 95% CI 1.02–5.94, *p* = 0.046). The events-per-variable (EPV) ratio for the multivariable Cox model was 5.3.

**Figure 2:**
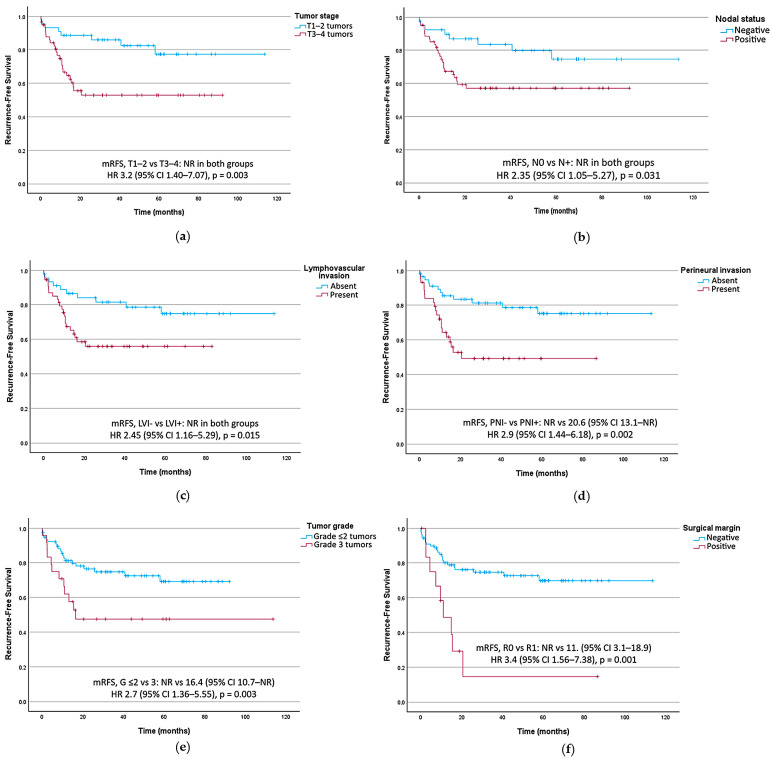
Recurrence-free survival curves stratified by key prognostic factors. (**a**) T TNM category. (**b**) Lymph node status. (**c**) Tumoral lymphovascular invasion (LVI). (**d**) Tumoral perineural invasion (PNI). (**e**) Histological tumor grade (G). (**f**) Surgical resection margin. CI, confidence interval; HR, hazard ratio; mRFS, median recurrence-free survival; NR, not reached.

All RFS analysis is shown in Table 2. Figure 2 presents RFS curves stratified by key prognostic factors in the univariate analysis.

#### 3.2.3. Recurrence-Free Survival by Histological Subtype

The intestinal subtype showed a trend toward improved RFS compared with the pancreaticobiliary subtype, although the difference was not statistically significant (HR 0.78, 95% CI 0.4–1.7, *p* = 0.54).

Adverse prognostic factors identified in the overall cohort were subsequently examined according to histological subtype. No consistent differences were observed between the intestinal and pancreaticobiliary subtypes. Subgroup analyses were limited by small and unbalanced sample sizes; in particular, grade 3 tumors were infrequent in the pancreaticobiliary subtype (4.3%), compared with the intestinal subtype (15.8%), precluding meaningful subgroup comparisons (see Supplementary Table S1).

#### 3.2.4. Impact of Adjuvant Therapy on Recurrence-Free Survival

In unadjusted analyses, use of adjuvant therapy was associated with a non-significant trend toward improved RFS in the overall cohort (HR 0.71, 95% CI 0.40–1.50, *p* = 0.35). Given the non-random allocation of adjuvant therapy and its association with higher-risk disease features, a multivariable Cox regression model adjusted for lymph node status and surgical resection margin status was performed. After adjustment, use of adjuvant therapy was significantly associated with improved RFS (HR 0.36, 95% CI 0.17–0.78; *p* = 0.009). Exploratory analyses including histological subtype as a covariate did not suggest differential associations between adjuvant therapy and outcomes.

### 3.3. Overall Survival in Localized Disease

#### 3.3.1. Median Overall Survival and Survival Rates

**Figure 3:**
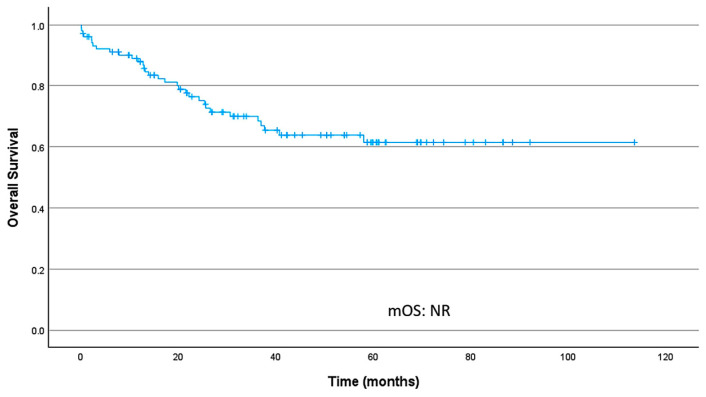
Overall survival curve in localized disease; median overall survival (mOS) was not reached.

Median overall survival (mOS) was not reached (95% CI, NR–NR). OS rates at 24 and 36 months were 76.4% (95% CI 68.2–85.6) and 70.1% (95% CI 61.1–80.3), respectively. Figure 3 presents the OS curve in localized disease.

#### 3.3.2. Prognostic Factors for Overall Survival

**Figure 4:**
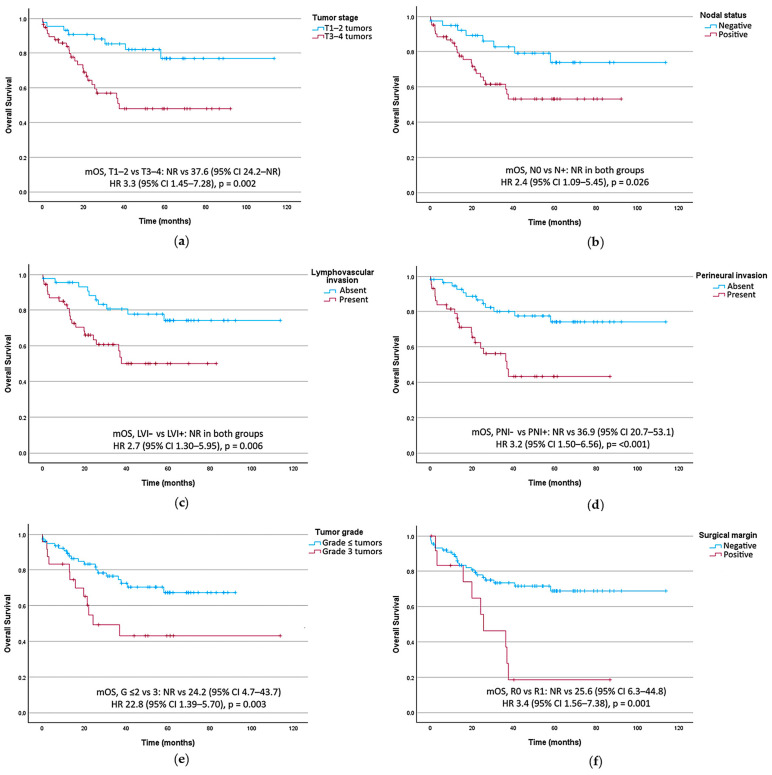
Overall survival curves stratified by key prognostic factors. (**a**) T TNM category. (**b**) Lymph node status. (**c**) Tumoral lymphovascular invasion (LVI). (**d**) Tumoral perineural invasion (PNI). (**e**) Histological tumor grade (G). (**f**) Surgical resection margin. CI, confidence interval; HR, hazard ratio; mRFS, median recurrence-free survival; mOS, Median overall survival; NR, not reached.

Worse OS was associated with T3–T4 tumors (HR 3.3, 95% CI 1.45–7.28, *p* = 0.002), lymph node-positive disease (HR 2.4, 95% CI 1.09–5.45, *p* = 0.026), lymphovascular invasion (HR 2.7, 95% CI 1.30–5.95, *p* = 0.006), perineural invasion (HR 3.2, 95% CI 1.50–6.56, *p* < 0.001), grade 3 tumors (HR 2.8, 95% CI 1.39–5.70, *p* = 0.003), and R1 resection margins (HR 3.4, 95% CI 1.56–7.38, *p* = 0.001), as shown in Table 3. Figure 4 presents OS curves stratified by key prognostic factors in the univariate analysis.

An increase in perioperative serum tumor marker levels was not associated with OS (CEA: HR 0.73, 95% CI 0.22–2.43, *p* = 0.614; CA 19-9: HR 1.38, 95% CI 0.69–2.76, *p* = 0.362).

In multivariate analysis, no variables remained independently associated with OS (Table 3). The EPV ratio for the multivariable Cox model was 5.3.

#### 3.3.3. Overall Survival by Histological Subtype

OS did not differ significantly between the intestinal and pancreaticobiliary histological subtypes (HR 0.83, 95% CI 0.40–1.70, *p* = 0.625). Subtype-specific analyses of prognostic factors did not show consistent differences; however, these analyses were limited by small and unbalanced sample sizes, as illustrated by the unequal distribution of tumor grade (15.8% in the intestinal subtype vs. 4.3% in the pancreaticobiliary subtype) and resection margin status (10.5% vs. 17.4%, respectively), precluding meaningful subgroup comparisons.

#### 3.3.4. Impact of Adjuvant Therapy on Overall Survival

In unadjusted analyses, use of adjuvant therapy was not significantly associated with overall survival (OS) (HR 0.71, 95% CI 0.35–1.45; *p* = 0.35). Consistent with the RFS analysis, after adjustment for lymph node status and surgical resection margin status, use of adjuvant therapy was significantly associated with improved OS (HR 0.40, 95% CI 0.19–0.85, *p* = 0.018). Exploratory analyses did not suggest differential associations between adjuvant therapy and OS according to histological subtype.

#### 3.3.5. Survival Analysis in Metastatic Disease

In the metastatic setting, which included both patients with metastatic disease ab initio and those who developed metastases after curative-intent treatment, the mOS was 13.6 months (95% CI, 10.9–16.3). Among patients receiving first-line systemic therapy, mOS was 11.8 months (95% CI, 8.1–15.5), and the median duration of first-line treatment was 5.8 months (range, 1.0–15.5).

For first-line treatment, median PFSmet was 6.7 months (95% CI, 1.6–11.8). 5 patients received second-line treatment, whereas none received third-line systemic therapy.

## 4. Discussion

### 4.1. Prognostic Factors

In our cohort, T3–T4 tumors, lymph node involvement, lymphovascular invasion, perineural invasion, high-grade histology (grade 3), and R1 resection margin were associated with both RFS and OS. However, multivariate analysis revealed that only R1 resection margin remained independently associated with poorer RFS, while none of the other factors were independently associated with OS. These findings are consistent with previously published data identifying these pathological features as markers of aggressive disease biology and poor prognosis, although not all reports have identified the same set of prognostic factors [12]. For instance, Zhang X et al. (2022) [9] identified the T TNM category and lymphovascular invasion as the independent prognostic factors for recurrence and OS after curative resection. In another retrospective study, only lymph node status and surgical resection margin were independent predictors of poor prognosis, whereas T3–T4 tumors, positive resection margin, lymph node involvement, lymphovascular and perineural invasion, and poor histological grade were associated with OS in univariate analysis [10]. A meta-analysis by Luchini et al. (2019) [13], which included 2379 patients, demonstrated that perineural invasion was strongly associated with both poorer survival and an increased risk of recurrence, further underscoring its prognostic significance. Similarly, Buyuktalanci et al. (2025) [14] reported that lymphovascular invasion and positive resection margin were independent prognostic markers, reinforcing the impact of these pathological features on patient outcomes. In contrast, Vilhordo et al. (2021) [11] found that lymph node involvement was the only independent prognostic factor in their series. Taken together, these variations may reflect differences in sample size, patient selection, histopathologic assessment, and treatment strategies across institutions and study periods. Nonetheless, the overall convergence of evidence highlights the multifactorial nature of prognosis in AC and the importance of comprehensive pathologic evaluation and multidisciplinary management, particularly for patients with high-risk features who may benefit from more aggressive adjuvant approaches.

Regarding histological subtypes, our study did not identify a statistically significant difference in survival outcomes between the intestinal and pancreaticobiliary types of AC, which contrasts with several previously published retrospective studies, including multicenter cohorts, registry data, and meta-analyses, reporting poorer prognosis for the pancreaticobiliary subtype [3,12,15,16]. These studies often attribute this disparity to more aggressive tumor biology, higher rates of lymph node involvement, and more advanced stage at diagnosis in the pancreaticobiliary group [3,12,15,16]. Despite the absence of statistical significance in our findings, the established medical literature supports considering histological subtype as a potentially relevant factor in risk stratification and treatment planning, particularly when integrated with other prognostic indicators.

In our cohort, the presence of obstructive jaundice at diagnosis showed a trend toward improved prognosis, although this did not reach statistical significance. This might be explained better by earlier detection and a higher likelihood of curative resection among jaundiced patients, rather than a protective biological effect of jaundice itself [5].

### 4.2. Adjuvant Treatment

Regarding adjuvant treatment, unadjusted analyses did not demonstrate a statistically significant association with outcomes. After adjustment for lymph node status and surgical resection margin, use of adjuvant therapy was associated with improved RFS and OS in our cohort. Evidence for adjuvant therapy in ampullary adenocarcinoma remains mixed. The European Study Group for Pancreatic Cancer (ESPAC)-3 trial, a randomized phase III multicenter trial of patients with periampullary cancer (of whom 69% had AC), failed to demonstrate an OS benefit for routine adjuvant chemotherapy in the overall study population. However, multivariate analysis adjusting for age, tumor grade, and lymph node status revealed a statistically significant survival benefit [17]. Aligned with these findings, a meta-analysis performed in 2017 by Acharya et al. [18], including 1671 patients who underwent curative surgery for periampullary adenocarcinoma, showed no significant survival benefit for adjuvant chemotherapy or chemoradiotherapy compared with observation, and ampullary-specific outcomes were not separately reported. Several other retrospective studies also did not find a significant association between adjuvant treatment and improved OS or disease-free survival. For example, in a multicenter retrospective study of 357 patients with AC, adjuvant chemotherapy (fluorouracil or gemcitabine-based) with or without radiotherapy was not associated with improved long-term OS after curative resection [19]. Similarly, Kang et al. (2022) [20], in a propensity score–matched analysis of 475 patients, reported no significant differences in OS or RFS between those receiving 5-FU plus leucovorin adjuvant chemotherapy and those managed with observation alone [20].

By contrast, a meta-analysis by Vo et al. (2021) [21], including 3538 patients from 27 studies, found that adjuvant therapy after curative resection significantly reduced mortality, with the greatest benefit seen for chemoradiotherapy. The benefit was most pronounced in high-risk patients (tumor invasion beyond the ampulla, positive resection margins, poor differentiation, or lymph node metastasis) and those with pancreaticobiliary subtype, whereas no clear benefit was observed in low-risk cases (confined to the ampulla, negative margins, well-to-moderately differentiated histology, or absence of nodal involvement) or in intestinal-subtype tumors. Notably, adjuvant treatment was not significantly associated with improved disease-free survival. In a large national cohort study, Nassour et al. (2018) [22] analyzed 4190 patients with resected AC and found that both adjuvant chemotherapy and chemoradiotherapy were associated with improved OS compared to observation, with the survival benefit particularly notable in patients with higher tumor and nodal stages. Similarly, a large German population-based study including 830 patients demonstrated that adjuvant chemotherapy significantly improves OS and disease-free survival in patients with advanced-stage disease, including T3–T4 tumors, lymph node metastases, and lymphovascular invasion [23]. Nevertheless, the optimal adjuvant approach remains unclear, as no head-to-head trials have directly compared chemotherapy with chemoradiotherapy in AC.

The prospective ADAPTA trial (NCT06068023, 2023) is a phase II study that is currently evaluating tailored adjuvant chemotherapy in resected AC, using CAPOX for intestinal and FOLFIRINOX for pancreaticobiliary or mixed subtypes, aiming to improve disease-free survival and provide prospective data to guide treatment strategies [24].

Current National Comprehensive Cancer Network (NCCN) guidelines for ampullary adenocarcinoma (Version 2.2025) recommend adjuvant chemotherapy largely based on extrapolation from colorectal, pancreatic, and biliary tract cancer data, in combination with expert consensus, reflecting the paucity of prospective evidence specific to ampullary cancers [25]. No guidelines have yet been published by the European Society for Medical Oncology (ESMO) or the American Society of Clinical Oncology (ASCO).

### 4.3. Advanced Disease

In unresectable locally advanced or metastatic AC, as with adjuvant treatment, there is a lack of randomized phase III trials specifically addressing systemic therapy. The ABC-02 trial (2010), which included a small proportion of patients with AC, demonstrated improved OS with cisplatin plus gemcitabine compared with gemcitabine alone in first-line advanced biliary tract cancer [26]. Similarly, the ABC-06 trial (2021), enrolling patients with advanced biliary tract cancers, including AC, reported a modest benefit of FOLFOX over active symptom control in the second-line setting [27]. Nevertheless, no studies have reported OS or progression-free survival (PFS) outcomes specifically for AC, and there remains no consensus on the optimal systemic chemotherapy regimen for this population. In practice, treatment recommendations are therefore extrapolated from phase III trials in colorectal, pancreatic, and biliary tract cancers, with guidance based on histologic subtype. For pancreaticobiliary or mixed subtypes, recommendations are based on pancreatic and biliary tract cancer trials, whereas for the intestinal subtype, they are derived from colorectal cancer trials [25].

### 4.4. Future Perspectives

The role of molecular profiling is becoming increasingly important, particularly for identifying candidates for targeted therapies and immunotherapy. Although prospective trials specific to AC are lacking, these patients are often considered for tumor-agnostic therapies based on extrapolated evidence, as they were largely excluded from most basket studies. Notably, the KEYNOTE-158 trial, a nonrandomized, open-label, multicenter phase II study, evaluated pembrolizumab in previously treated advanced solid tumors with microsatellite instability–high (MSI-H) or mismatch repair deficiency (dMMR), demonstrating durable responses across tumor types and supporting its tumor-agnostic approval [28,29]. The DESTINY PanTumor 02 trial, a multicenter phase II basket study, evaluated trastuzumab deruxtecan in patients with HER2 IHC 3+ solid tumors across multiple histologies, demonstrating robust and durable antitumor activity and providing pivotal evidence for its tumor-agnostic Food and Drug Administration (FDA) approval [30]. The NAVIGATE and STARTRK programs showed that tropomyosin kinase receptor inhibitors (larotrectinib and entrectinib, respectively) produce robust and durable responses in patients with neurotrophic receptor tyrosine kinase (NTRK) gene fusion tumors across diverse tumor types, supporting their tumor-agnostic approvals [31,32].

These data support current NCCN guidelines recommending comprehensive molecular testing in unresectable or metastatic AC [25]. More recently, a retrospective study by Fabregat-Franco et al. (2025) [8] showed that over half of AC harbor potentially actionable molecular alterations, most frequently in ERBB2 and DNA repair pathways. This finding highlights the feasibility of precision-oncology approaches in this rare malignancy and underscores the need for prospective, biomarker-driven trials.

Furthermore, the integration of multi-omic profiling, patient-derived models, and liquid biopsy approaches may enhance risk stratification, enable real-time monitoring of minimal residual disease, and support truly personalized therapeutic strategies. Ultimately, collaborative efforts and international registries will be essential to translate these advances into improved patient outcomes.

### 4.5. Study Limitations

This study is limited by its retrospective design, small sample size, and heterogeneity of treatments, which may affect statistical power and generalizability. In addition, the limited number of events resulted in a modest EPV ratio in multivariable analyses, potentially affecting model stability and limiting the detection of independent prognostic factors. Given these constraints, more complex modeling strategies and formal sensitivity analyses were not undertaken, and multivariable findings should be interpreted as exploratory and hypothesis-generating rather than definitive.

## 5. Conclusions

Among the prognostic factors evaluated, several variables were associated with outcomes in univariate analyses; however, R1 resection margin status emerged as the only independent prognostic factor for RFS, but not for OS, in multivariable analysis. No consistent differences in RFS or OS were observed according to histological subtype. While unadjusted analyses did not demonstrate a significant association between adjuvant therapy and outcomes, exploratory adjusted analyses accounting for lymph node status and surgical resection margin suggested an association with improved survival, which should be interpreted with caution given the retrospective design and limited sample size. In the metastatic setting, survival outcomes were descriptively characterized.

Given the inherent limitations of this single-center retrospective cohort, subgroup analyses were exploratory and underpowered, and no definitive conclusions can be drawn. Larger, preferably multicenter studies are warranted to better define prognostic markers and clarify the role of adjuvant therapy in AC.

From a clinical perspective, these findings highlight surgical margin optimization and high-quality pathological reporting as actionable targets in routine practice.

## Figures and Tables

**Table 1:** Baseline clinical, demographic, and pathological characteristics of patients with ampullary carcinoma.

Characteristics	Number of Patients, *n* = 106 (100%)
**Demographics**	
**Age (years)**	
Median	69
IQR	63–77
**Sex**	
Male	62 (58.5)
Female	44 (41.5)
**ECOG** PS	
0	44 (41.5)
1	54 (50.9)
≥2	8 (7.5)
**Clinical characteristics**	
**Presenting symptoms**	
Obstructive jaundice	91 (85.8)
Weight loss	44 (41.5)
Abdominal pain	27 (25.5)
Nausea and vomiting	22 (20.8)
**Serum tumor markers**	
CA 19-9 > 34 (U/mL)	50 (47.2)
CEA > 5 (ng/mL)	14 (13.2)
**Clinical stage of diagnosis**	
I	30 (28.3)
II	7 (6.6)
III	65 (61.3)
IV	4 (3.8)
**Tumor characteristics**	
**Tumor histological subtype**	
Pancreaticobiliary	48 (45.3)
Intestinal	38 (35.8)
Mixed	20 (18.9)
**Clinical T category**	
cT1	14 (13.2)
cT2	31 (29.2)
cT3	51 (48.1)
cT4	10 (9.4)
**Clinical lymph node status**	
Metastatic lymph node	65 (61.3)
Non-metastatic lymph node	41 (38.7)
**Histological grade**	
1	27 (25.5)
2	51 (41.1)
3	28 (26.4)
Surgery and postoperative characteristics *	102 (96.2) *
**Pancreaticoduodenectomy**	102 (100)
**Tumor histological subtype**	
Pancreaticobiliary	46 (45.1)
Intestinal	38 (37.3)
Mixed	18 (17.6)
**Surgical resection margins**	
Positive	13(12.7)
Negative	89 (87.3)
**Pathological T category**	
pT3–4	59 (57.8)
pT1–2	43 (42.2)
**Pathological lymph node status**	
Metastatic lymph node	62 (60.7)
Non-metastatic lymph node	40 (39.3)
**Histological grade**	
1	19 (18.6)
2	58 (56.9)
3	25 (24.5)
**Tumoral lymphovascular invasion**	
Present	55 (53.9)
Absent	47(46.1)
**Tumoral perineural Invasion**	
Present	44 (43.1)
Absent	58 (56.9)
**Neoadjuvant chemotherapy**	1 (1.0) *
FOLFIRINOX	1 (1.0)
**Adjuvant chemotherapy**	39 (38.2) *
Gemcitabine	14 (13.7)
Gemcitabine/capecitabine	10 (9.8)
Capecitabine	5 (4.9)
FOLFOX	2 (2.0)
CAPOX	2 (2.0)
FOLFIRINOX	1 (1.0)
GEMOX	1 (1.0)
Missing	4 (3.9)
**Adjuvant chemoradiotherapy**	9 (8.8) *
Capecitabine	9 (8.8)
**Recurrence**	102 (100.0) *
Yes	27 (26.5)
No	75 (73.5)
**Recurrence patterns**	34 (33.3) *
Local	1 (1.0)
Hepatic	18 (17.4)
Lymph node	6 (5.9)
Pulmonary	4 (3.9)
Peritoneal	4 (3.9)
Bone	1 (1.0)
**First-line systemic treatment after recurrence**	27 (26.5) *
FOLFIRINOX	3 (2.9)
Gemcitabine	5 (4.9)
Capecitabine	1 (1.0)
Gemcitabine-capecitabine	1 (1.0)
Cisplatin-gemcitabine	3 (2.9)
Gemcitabine-nab-paclitaxel	2 (2.0)
CAPOX	1 (1.0)
FOLFOX	3 (2.9)
5-fluorouracil	1 (1.0)
Best supportive care	7 (6.9)
**Second-line systemic treatment**	5 (4.9) *
Gemcitabine-capecitabine	1 (1.0)
Gemcitabine	1 (1.0)
FOLFIRINOX	1 (1.0)
FOLFIRI	1 (1.0)
Best supportive care	1 (1.0)

CA 19-9—Carbohydrate antigen 19-9; CEA—Carcinoembryonic antigen; CAPOX—capecitabine + oxaliplatin; FOLFIRI—5-fluorouracil + leucovorin + irinotecan; ECOG PS—Eastern Cooperative Oncology Group performance status; FOLFOX—5-fluorouracil + leucovorin + oxaliplatin; FOLFIRINOX—5-fluorouracil + leucovorin + irinotecan + oxaliplatin; GEMOX—gemcitabine + oxaliplatin. * Percentages from surgery and postoperative characteristics onward are calculated based on the 102 patients who underwent surgery.

**Table 2:** Prognostic factors for recurrence-free survival: univariate and multivariate analysis.

	Univariate Analysis		Multivariate Analysis	
	HR (95% CI)	*p*-Value	HR (95% CI)	*p*-Value
**T3–T4 tumors**	3.2 (1.40–7.07)	0.003	1.7 (0.60–4.96)	0.317
**Metastatic lymph node**	2.35 (1.05–5.27)	0.031	1.3 (0.51–3.46)	0.545
**Tumoral lymphovascular invasion**	2.45 (1.16–5.29)	0.015	1.4 (0.59–3.45)	0.437
**Tumoral perineural invasion**	2.9 (1.44–6.18)	0.002	1.1 (0.37–3.1)	0.899
**Histological grade 3**	2.7 (1.36–5.55)	0.003	1.8 (0.77–3.72)	0.193
**R1 resection margin**	4.06 (1.85–8.92)	<0.001	2.5 (1.02–5.93)	0.046
**Intestinal histological subtype**	0.78 (0.4–1.7)	0.54	N/A	N/A
**Obstructive jaundice**	0.85 (0.30–2.40)	0.77	N/A	N/A
**Elevated perioperative serum CEA**	0.77 (0.23–2.56)	0.853	N/A	N/A
**Elevated perioperative serum CA 19-9**	1.46 (0.72–2.92)	0.282	N/A	N/A

CI—confidence interval; HR—hazard ratio; N/A—Not applicable.

**Table 3:** Prognostic factors for overall survival: univariate and multivariate analysis.

	Univariate Analysis		Multivariate Analysis	
	HR (95% CI)	*p*-Value	HR (95% CI)	*p*-Value
**T3–T4 tumors**	3.3 (1.45–7.28)	0.002	1.8 (0.61–5.43)	0. 284
**Metastatic lymph node**	2.4 (1.09–5.45)	0.026	1.2 (0.43–3.14)	0.773
**Tumoral lymphovascular invasion**	2.7 (1.30–5.95)	0.006	1.6 (0.68–3.95)	0.269
**Tumoral perineural invasion**	3.2 (1.50–6.56)	<0.001	1.2 (0.40–3.41)	0.775
**Histological grade 3**	2.8 (1.39–5.70)	0.003	1.8 (0.82–3.87)	0.141
**R1 resection margin**	3.4 (1.56–7.38)	0.001	1.9 (0.77–4.47)	0.168
**Intestinal histological subtype**	0.83 (0.40–1.70)	0.625	N/A	N/A
**Obstructive jaundice**	0.85 (0.29–2.45)	0.770	N/A	N/A
**Elevated perioperative serum CEA**	0.73 (0.22–2.43	0.614	N/A	N/A
**Elevated perioperative serum CA 19-9**	1.38 (0.69–2.76)	0.362	N/A	N/A

CI—confidence interval; HR—hazard ratio; N/A—Not applicable.

## Data Availability

The original contributions presented in this study are included in the article. Further inquiries can be directed to the corresponding author.

## Supplementary Materials

The following supporting information can be downloaded at: https://www.mdpi.com/article/10.3390/cancers18040707/s1, Table S1: Exploratory analyses of prognostic factors according to histological subtype.

## Abbreviations

The following abbreviations are used in this manuscript:

AC	Ampullary carcinoma
ASCO	American Society of Clinical Oncology
CA 19-9	Carbohydrate antigen 19-9
CAPOX	Capecitabine + oxaliplatin
CEA	Carcinoembryonic antigen
CI	Confidence interval
dMMR	Mismatch repair deficiency
DNA	Deoxyribonucleic acid
ECOG PS	Eastern Cooperative Oncology Group performance status
ESMO	European Society for Medical Oncology
EPV	Events-per-variable
FOLFIRINOX	5-fluorouracil + leucovorin + irinotecan + oxaliplatin
FOLFOX	5-fluorouracil + leucovorin + oxaliplatin
GEMOX	Gemcitabine + oxaliplatin
GI	Gastrointestinal
HR	Hazard ratio
IQR	Interquartile range
MSI-H	Microsatellite instability–high
mOS	Median overall survival
mPFS	Median progression-free survival
NCCN	National Comprehensive Cancer Network
NR	Not reached
NTRK	Neurotrophic receptor tyrosine kinase
OS	Overall survival
PFS	Progression-free survival
PFSmet	Progression-free survival in the metastatic setting
RFS	Recurrence-free survival
TMB	Tumor mutational burden
TNM	Tumor, Nodes, Metastasis
