# Supplementary material for: Ampullary Carcinoma: Prognostic Factors and a Literature Review
Source: Cancers (Basel). 2026 Feb 22;18(4):707. doi: 10.3390/cancers18040707 (PMC12939735; doi:10.3390/cancers18040707)
Supplement: Supplementary file 1 [file cancers-18-00707-s001.zip › cancers-4147124-supplementary.pdf]

Supplementary Table S1. Exploratory analyses of prognostic factors according to histological subtype

	Outcome	Tumor subtype: INT (median, months, 95% CI)	Tumor subtype: PB (median, months, 95% CI)	HR (95% CI)*	*p*-Value
T3–T4 tumors	RFS	NR (12.1–NR)	20.6 (11.0–NR)	1.6 (0.62–4.24)	0.315
	OS	NR (21.5–NR)	36.3 (20.0–NR)	1.8 (0.69–4.80)	0.212
Metastatic lymph node	RFS	10.2 (8.3–NR)	NR (16.4–NR)	0.7 (0.27–1.83)	0.464
	OS	39.6 (15.5–NR)	NR (24.7–NR)	0.8 (0.32–2.16)	0.701
Tumoral lymphovascular invasion	RFS	NR (10.2–NR)	NR (15.4–NR)	1.1 (0.42–2.88)	0.859
	OS	NR (15.8–NR)	36.9 (24.2–NR)	1.1 (0.42–2.94)	0.825
Tumoral perineural invasion	RFS	NR (10.2–NR)	20.6 (14.9–NR)	1.1 (0.44–2.57)	0.887
	OS	37.6 (21.5–NR)	36.3 (24.2–NR)	1.2 (0.50–2.93)	0.655
Histological grade 3	RFS	6.4 (2.1-NR)	NR (16.4-NR)	0.1 (0.025-0.52)	< 0.001
	OS	14.5 (2.1-NR)	NR (24.2-NR)	0.1 (0.03-0.57)	0.001
R1 resection margin	RFS	9.6 (9.6–NR)	14.9 (7.3–NR)	1.5 (0.37–5.98)	0.540
	OS	37.6 (37.6–NR)	25.6 (20.0–NR)	8.7 (0.99–76.43)	0.023

CI - confidence interval; HR – hazard ratio; INT – Intestinal; OS – overall survival; PB – Pancreaticobiliary; RFS – recurrence-free survival. *HR < 1 indicates higher risk in the intestinal subtype; HR > 1 indicates higher risk in the pancreaticobiliary subtype.

These subgroup analyses are exploratory and based on small numbers. Hazard ratios should be interpreted with caution and are not intended for inferential purposes.

These analyses are exploratory and based on very small subgroup sizes. Hazard ratio estimates should be interpreted with caution and are not intended for inferential purposes.
